# Enterokinase deficiency associated with novel TMPRSS15 gene mutations: a case report

**DOI:** 10.3389/fped.2025.1484208

**Published:** 2025-01-29

**Authors:** Yunxi Li, Ruijuan Li, Yanyan Pan, Weiran Zhou, Xingcui Wang, Linlin Dong, Xuemei Liu, Hongxia Zhang

**Affiliations:** Department of Pediatric Nephrology and Rheumatism and Immunology, Children’s Hospital Affiliated to Shandong University, Jinan Children’s Hospital, Jinan, China

**Keywords:** enterokinase deficiency, TMPRSS15, gene, children, case report

## Abstract

**Background:**

Enterokinase deficiency (EKD,OMIM #226200) is a rare autosomal recessive genetic disorder caused by mutations in transmembrane protease serine 15 (TMPRSS15). Herein, we report a case of EKD in a patient with novel compound heterozygous TMPRSS15 mutations.

**Case presentation:**

A 2-month-old female infant presented with chronic diarrhea, vomiting, pallor, general edema, skin lesions, and a failure to gain weight. Further examination revealed anemia, hypoalbuminemia, and multiorgan damage. Whole-exome sequencing further revealed two novel heterozygous variants of TMPRSS15: c.2611C>T (p.Arg871Ter) and c.1584_1585insCTTT (p.Glu529LeufsTer2). The clinical symptoms dramatically improved following pancreatic enzyme replacement. During a one-year follow-up, the patient showed a normal rate of physical development, with no recurrence of anemia, hypoproteinemia, coagulopathy or skin lesions.

**Conclusion:**

Herein, we presented a clinical case of EKD with two novel compound heterozygous mutations in TMPRSS15 who achieved dramatic symptom improvements following pancreatic enzyme supplementation. This case enriches the genotypic spectrum of EKD and provides a reference for the diagnosis and treatment of similar cases. This case suggests that if zymogen activation testing is not possible, genetic analysis may be an effective tool to facilitate early diagnosis. Further, early pancreatic enzyme supplementation is a clinical strategy which can achieve satisfactory outcomes.

## 1 Introduction

Enterokinase, also known as enteropeptidase, is a proteolytic enzyme secreted by the duodenum. Enterokinase catalyzes the conversion of trypsinogen into active trypsin, which in turn activates other pancreatic zymogens such as chymotrypsinogen and procarboxypeptidases (1). As such, the protein digestion process largely depends on the activity of enterokinase. Enterokinase deficiency (EKD, OMIM #226200) is a rare autosomal recessive genetic disease caused by mutations in the transmembrane protease serine 15 (TMPRSS15) gene. EKD in children is characterized by chronic diarrhea, hypoproteinemia, anemia, and failure to thrive. Fortunately, the prognosis of EKD is good provided that an early diagnosis is achieved, allowing appropriate enzyme supplementation. The first case of EKD was reported by Hadorn et al. in 1969 (2). However, in the intervening decades, only 13 cases have been reported (2–15) (Table 1). Herein, we report a case of EKD with novel compound heterozygous mutations in TMPRSS15 (c.2611C>T; c.1584_1585insCTTT).

**Table 1:** Summary of reported cases of enterokinase deficiency.

Case	Mutation	Type	Domain affected	Clinical presentation	Reference
1	c.2611C > T(p.Arg871Ter)	Nonsense	Serine Protease	Diarrhea, vomiting, anemia, edema, hypoalbuminemia, failure to thrive, skin lesions, multiple organ dysfunctions	Current study
	c.1584_1585insCTTT(p.Glu529LeufsTer2)	Frameshift	C1r/s		
2	c.1216C > T(p.Arg406Term)	Nonsense	MAM	Diarrhea, vomiting, anemia, edema, hypoalbuminemia, failure to thrive, skin lesions	Chen et al. (8)
3	c.1350dupA(p.Val451SerfsTer21)	Frameshift	MAM	Diarrhea,edema, hypoalbuminemia, anemia, hepatomegaly, failure to thrive	Madhusudan et al. (7)
	c.3004G > A(p.Gly1002Arg)	Missense	Serine Protease		
4	c.1921G > A(p.Glu641Lys)	Missense	LDLR	Diarrhea, vomiting, hypoalbuminemia, failure to thrive, infection, died when he was 2 years old.	Wang et al. (12)
	c.2396T > A(p. Val799Asp)	Missense	Serine Protease		
5	ND	ND	ND	Diarrhea, failure to thrive, arrhythmia	Marshall et al. (15)
6	ND	ND	ND	Diarrhea, edema, hypoalbuminemia, anemia, failure to thrive, mild liver damage, abnormal coagulation function	Green et al. (10)
7	ND	ND	ND	Diarrhea, edema, hypoalbuminemia, failure to thrive, skin lesions	Ghishan et al. (6)
8	ND	ND	ND	Diarrhea, vomiting, anemia, edema, hypoalbuminemia, failure to thrive	Follet et al. (9)
9–10	c.2135C > G(p.Ser712Term)	Nonsense	MSCR	Occurrence in two siblings: Boy: Diarrhea, vomiting, edema, anemia, hypoalbuminemia, failure to thrive, electrolyte disturbance, sepsis; Girl:Diarrhea, vomiting, anemia, hemolysis	Hadorn et al. (5) Holzinger et al. (14)
	c.2569C > T(p.Arg857Term)	Nonsense	Serine Protease		
11	c.781C > T(p.Gln261Term)	Nonsense	C1r/s	Diarrhea, vomiting, edema, anemia, hepatomegaly, hypoalbuminemia, failure to thrive, celiac disease (reported when he was 40 years old)	Haworth et al. (4) Moroz et al. (11) Holzinger et al. (14)
	c.2707_2708delGT	Frameshift	Serine Protease		
12	ND	ND	ND	Diarrhea, edema, hypoalbuminemia, anemia, failure to thrive	Polonovski et al. (13)
13	ND	ND	ND	Diarrhea, anemia, failure to thrive	Tarlow et al. (3)
14	ND	ND	ND	Diarrhea, edema, hypoalbuminemia, anemia, failure to thrive	Tarlow et al. (3) Hadorn et al. (2)

LDLR, LDL receptor–like domain; C1r/s, complement component C1r-like domain; MAM, meprin-like domain; MSCR, macrophage scavenger receptor-like domain; ND, not done.

## 2 Case presentation

A 2-month-old female infant was admitted to hospital due to pallor and edema lasting 6 days. She also experienced a slight cough, rash, diarrhea, and occasional vomiting. Diarrhea was described as the passage of loose green stools seven to eight times per day, at 1 month of age. The infant was born at term and weighed 4.15 kg, and was consistently breastfed after birth. Her parents were healthy non-consanguineous Chinese adults, and the patient had no relevant family history. On examination, the infant weighed 4.9 kg, indicating a weight gain of only 0.75 kg in the first 2 months of life. The patient was pale and puffy, with generalized edema. Hazel rashes, erosions, and crusts were observed on the neck and lower extremities.

The initial laboratory investigations revealed anemia (Hb 51 g/L), hypoalbuminemia (Alb 16.4 g/L), coagulation abnormalities (PT 94.40s, APTT 82.70s, TT 54.20s, Fib 0.24 g/L, D-Di 0.19 mg/L), myocardial damage (CK-MB 26.02 ng/ml, CK 1117U/L, LDH 403U/L, AST 129U/L), and renal dysfunction (Cr 36 μmol/L). Her reticulocyte and bilirubin levels were also slightly elevated (Ret 5.32%, TBIL 19.8 μmol/L, DBIL 11.6 μmol/L, IBIL normal). Coombs test results were negative. Her white blood cell (WBC) count was 12.28 × 10^9^/L, with 57.2% neutrophils and 40.2% lymphocytes, while her platelet (PLT) count was 101 × 10^9^/L. The serum procalcitonin and ferritin levels were increased (PCT 1.86 ng/ml, Ferr 1020 μg/L). Further, her complement C3 level was decreased to 0.359 g/L, whereas that of C4 was normal. Here C-reactive protein (CRP), erythrocyte sedimentation (ESR), alanine aminotransferase (ALT), antinuclear antibody (ANA), IgA, IgG, IgM, K+, Na+, and Cl- concentrations were all within the normal range. Stool microscopy and fecal calprotectin levels revealed no abnormalities. Urinary protein/creatinine ratio (UPCR) was >0.2, while multiple urinalysis and microalbuminuria were normal, which may be related to the low urinary creatinine excretion rate, contributing to the overestimation of UPCR (16). Ultrasound (US) revealed echogenicity of the liver parenchyma; however, US images of the gallbladder, pancreas, kidney, and gastrointestinal tract all appeared normal. Cardiac ultrasonography indicated patent foramen ovale and a normal ejection fraction. Computed tomography further revealed fatty infiltration in the liver, with no abnormal changes in the lungs. Brain MRI was normal.

**Figure 1:**
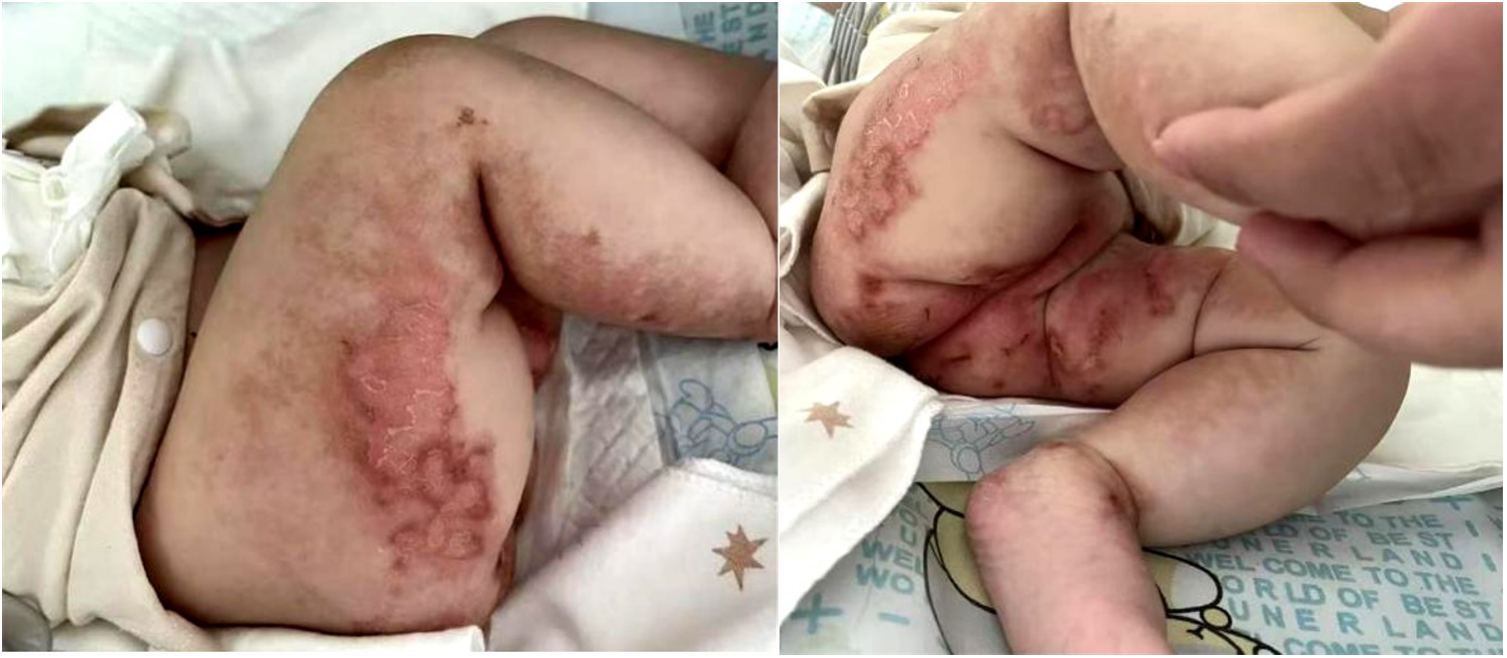
Erythema, with erosion, exudation and crust on the extremities.

After admission, the patient underwent surveillance with ECG-monitoring, and was treated with antibiotics, intravenous albumin, fresh frozen plasma, fibrinogen preparation, and red blood cell transfusions. Meanwhile, the patient was started on a new feeding regimen with a deep-hydrolyzed formula. On the fourth day of hospitalization, the patient's myocardial zymogram and creatinine levels returned to normal, and coagulation function gradually improved. However, diarrhea and vomiting were not alleviated. Regarding skin lesions, following consultation with attending neonatologists and dermatologists, the patients was suspected to have incontinentia pigmenti (PI) as the linear erythema, with erosion, exudation and crust on the extremities (Figure 1).

**Figure 2:**
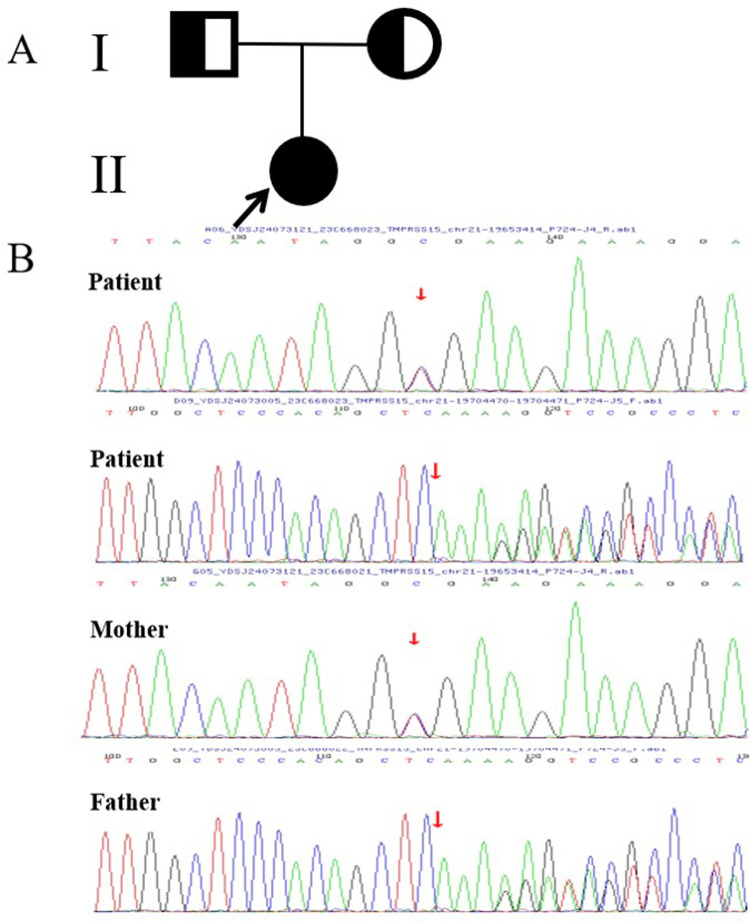
**(A)** The proband's family tree diagram; the arrow indicates the proband. The circles represent the proband and her mother, and the square represents her father. **(B)** Sanger sequencing diagram. The mutation was verified by Sanger sequencing, which revealed two novel compound heterozygous mutations in exons 22 [c.2611C > T (p.Arg871Ter)] and 14 [c.1584-1585insCTTT (p.Glu529LeufsTer2)] of TMRRSS15 gene, which were inherited from the patient's mother and father, respectively.

Based on the above results, we ruled out nephrotic syndrome, serious liver dysfunction, heart failure, chronic infection, malignant tumor, intestinal lymphangiectasia and connective tissue disease as potential diagnoses. However, PI, congenital diarrhea, immune deficiency were retained for consideration. Gastrointestinal endoscopy was recommended to further clarify the patient's etiology; however, her parents refused. Given the patient's sever presentation and broad differential diagnosis, peripheral blood samples were collected from the patient and her family members to perform whole-exome sequencing (WES) after obtaining written consent from the parents. The results showed that the patient had two novel compound heterozygous mutations in exons 22 [c.2611C > T (p.Arg871Ter)] and 14 [c.1584_1585insCTTT (p.Glu529LeufsTer2)] of the TMRRSS15 gene (Figure 2). Sanger sequencing confirmed that the variants were inherited from her parents. Her mother carried the c.2611C > T mutation and her father carried the c.1584_1585insCTTT mutation. According to the American College of Medical Genetics (ACMG) guidelines for variant classification, c.2611C > T and c.1584_1585insCTTT were classified as “likely pathogenic” variants. Evidence, including PVS1 (null variant) and PM2 (extremely low frequency in the population databases), is presented to support this classification.

Ultimately, the patient was diagnosed with EKD based on her clinical characteristics and genetic testing results. Pancreatic enzyme replacement therapy was performed immediately following a definitive diagnosis of EKD, resulting in dramatic improvements in diarrhea, vomiting, malnutrition, and skin lesions. The patient was subsequently fed on a deep-hydrolyzed formula, and continued to receive pancreatic enzyme supplementation. After a one year of treatment, the infant showed a normal rate of physical development, with no recurrence of anemia, hypoproteinemia, coagulopathy, or skin lesions.

## 3 Discussion

Enterokinase deficiency (EKD,OMIM #226200) is an extremely rare autosomal recessive disease caused by mutations in the TMPRSS15 gene (14), which is located on chromosome 21q21.1, and contains 25 exons (17, 18). To date, only twelve pathogenic variations have been described in the human Gene Mutation Database (HGMD®) and four in ClinVar (Tables 1, 2). Reported mutation types predominantly include nonsense and frameshift mutations. This gene encodes enterokinase, a serine protease secreted by the intestinal brush border. As a physiological activator, enterokinase first converts trypsinogen into active trypsin in the duodenum. Trypsin subsequently activates other proteolytic enzymes and the remaining trypsinogens. Aberrations in TMPRSS15 gene expression may result in a reduction or the abolition of the enzymatic function of enterokinase, leading to severe disturbances in protein digestion. In this case, the patient had two novel compound heterozygous mutations in exons 22 [c.2611C > T (p.Arg871Ter)] and 14 [c.1584_1585insCTTT (p.Glu529LeufsTer2)] of TMRRSS15, which resulted in the premature termination of Arg871 in the serine protease domain, and a frameshift of amino acids in the complement component C1r-like domain (C1/rs). Loss-of-function mutations in TMRRSS15 produce the EKD phenotype.

**Table 2:** Summary of other unreported mutations from HGMD and clinVar.

Mutation	Type	Domain affected	Clinical presentation	Reference
c.2486+1G>A	Splice mutation	NA	NA	NA
c.2294del (p.Leu765fs)	Frameshift	MSCR	NA	NA
c.1922_1G>A	Splice mutation	NA	NA	NA
c.1350dup (p.Val451fs)	Frameshift	MAM	NA	NA
c.1428+2T>G	Splice mutation	MAM	NA	NA
c.2325delA	Frameshift	Serine Protease	NA	NA
c.2294delT	Frameshift	MSCR	NA	NA
c.151_155delGCACT	Frameshift	NA	NA	NA
c.2808_2809insATCA	Frameshift	Serine Protease	NA	NA

MAM, meprin-like domain; MSCR, macrophage scavenger receptor-like domain; NA, not available.

Children with EKD typically develop symptoms such as chronic diarrhea, anemia, hypoproteinemia and undergrowth (2–13, 15), which was consistent with the presentation of our patient. Previous reports have elucidated the mechanisms underlying these conditions. The finding of diarrhea was likely attributable to secondary decreased activities of digestive enzymes, such as lipase, amylase, and disaccharidase (3, 5). Owing to general protein malabsorption, the patient subsequently presented with hypoalbuminemia and systemic protein loss. Anemia likely occurred due to impaired hemoglobin synthesis caused by protein deficiency. The observed hemolysis may be related to vitamin E deficiency (5, 10). The manifestation of fatty liver infiltration developed secondary to protein-energy malnutrition, which is similar to the phenotype observed in patients with severe malnutrition and kwashiorkor (4). In addition, coagulopathy with prolonged PT and APTT was likely caused by inadequate coagulation factors synthesis.

Skin lesions have been previously described in two cases: one with eczema around the perioral and diaper areas (6) and another with acrodermatitis enteropathica-like lesions (8). Our patient further developed skin damage, indicating that skin manifestations are likely to be a novel EKD phenotype. We initially considered a diagnosis of incontinentia pigmenti (IP), a rare X-linked dominant neuroectodermal disorder primarily caused by mutations in the IKBKG/NEMO gene, which may be associated with immunodeficiency, autoinflammatory and autoimmune diseases (e.g., inflammatory bowel disease, Behcet syndrome, systemic lupus erythematosus, etc.) (19–22). However, unlike IP, the patient's skin lesions did not following the four typical stages (erythema and vesicles, verrucous hyperplasia, hyperpigmentation, and atrophy), and was significantly improved after treatment with pancreatic enzyme replacement therapy. Furthermore, we found no abnormalities in the patient's neuroectodermal tissues, including the eyes, hair, teeth, and nails, or the central nervous system. Further, no pathogenic genetic variants that led to other illnesses were detected. Consequently, the diagnosis of IP was ruled out. In fact, skin manifestations could be seen in a variety of nutritional diseases, including cystic fibrosis and essential fatty acids deficiency, among others. In EKD, skin lesions may develop as a result of an interaction between proteins, microelements, and essential fatty acid deficiencies secondary to malabsorption.

Our patient also presented with multiple organ dysfunctions, including transient abnormalities in renal function, myocardial injury, and coagulation disturbances, which may be explained by infection, which has been reported to worsen EKD. Wang et al. (12). previously reported the case of a patient with EKD who developed intestinal and pulmonary infections during hospitalization, subsequently dying from this illness 2 weeks after suspending treatment. Nutrient malabsorption may contribute to immunocompromization, increasing the risk of infection and mortality. In the present case, multi-organ damage gradually improved following anti-infective therapy.

EKD is considered treatable. Enzyme replacement therapy greatly improves the prognosis of EKD, allowing patients to grow without obvious symptoms, sometimes even without enzyme supplementation. However, if treatment is delayed, patients may suffer from the severe long-term effects of malnutrition. In the past, EKD was diagnosed by assaying the enzymatic activity of the duodenal juice using the zymogen activation test (2). However, in practice, some families can be hesitant about the invasive procedure, particularly in infants. With the further in-depth study of the genetic mechanisms of the disease, genetic analysis can help to facilitate the early diagnosis of clinically clear single-gene genetic diseases, such as EKD, trypsin deficiency, cystic fibrosis, etc. This case indicates that EKD should be included in the differential diagnosis of children with clinical symptoms, such as chronic diarrhea, hypoproteinemia and failure to thrive, with no improvement following conventional treatment. In this case, although zymogen activation tests and endoscopy were not performed, the patient was diagnosed with EKD by WES, and showed dramatic improvements in symptoms and general health with enzyme supplementation. During the one-year follow-up, the patient showed a normal rate of physical development, with no recurrence of skin lesions or multisymptom damage.

## 4 Conclusion

Overall, in this report, we describe a patient with EKD with two novel compound heterozygous mutations in TMPRSS15 who achieved dramatic improvements in symptoms with pancreatic enzyme supplements. This case enriches the genotypic spectrum of EKD, and provides a reference for diagnosis and treatment. If zymogen activation testing is not possible, genetic analysis may be an effective tool to facilitate early diagnosis. Early pancreatic enzyme supplementation is a clinically substantial factor influencing satisfactory outcomes.

## Data Availability

The original contributions presented in the study are included in the article/Supplementary Material, further inquiries can be directed to the corresponding author.

## References

[1] Light A Janska H. Enterokinase (enteropeptidase): comparative aspects. Trends Biochem Sci. (1989) 14:110–2. 10.1016/0968-0004(89)90133-3 2658218

[2] Hadorn B Tarlow MJ Lloyd JK Wolff OH. Intestinal enterokinase deficiency. Lancet. (1969) 1:812–3. 10.1016/S0140-6736(69)92071-6 4180366

[3] Tarlow MJ Hadorn B Arthurton MW Lloyd JK. Intestinal enterokinase deficiency. A newly-recognized disorder of protein digestion. Arch Dis Child. (1970) 45:651–5. 10.1136/adc.45.243.651 5477679 PMC1647492

[4] Haworth JC Gourley B Hadorn B Sumida C. Malabsorption and growth failure due to intestinal enterokinase deficiency. J Pediatr. (1971) 78:481–90. 10.1016/S0022-3476(71)80231-7 4322674

[5] Hadorn B Haworth JC Gourley B Prasad A Troesch V. Intestinal enterokinase deficiency. Occurrence in two sibs and age dependency of clinical expression. Arch Dis Child. (1975) 50:277–82. 10.1136/adc.50.4.277 1147667 PMC1544443

[6] Ghishan FK Lee PC Lebenthal E Johnson P Bradley CA Greene HL. Isolated congenital enterokinase deficiency. Recent findings and review of the literature. Gastroenterology. (1983) 85:727–31. 10.1016/0016-5085(83)90033-1 6347801

[7] Madhusudan M Sankaranarayanan S Ravikumar T. Enterokinase deficiency: a case of pancreatic insufficiency. Indian J Pediatr. (2021) 88:825. 10.1007/s12098-021-03801-w 34106441

[8] Chen Y Li Z Liu C Wang S. Enterokinase deficiency with novel TMPRSS15 gene mutation masquerading as acrodermatitis enteropathica. Pediatr Dermatol. (2023) 40:389–91. 10.1111/pde.15197 36410965

[9] Follett GF Macdonald TH. Intestinal enterokinase deficiency. Acta Paediatr Scand. (1976) 65:653–5. 10.1111/j.1651-2227.1976.tb04948.x 961414

[10] Green JR Bender SW Posselt HG Lentze MJ. Primary intestinal enteropeptidase deficiency. J Pediatr Gastroenterol Nutr. (1984) 3:630–3. 10.1097/00005176-198409000-00026 6384461

[11] Moroz SP Hadorn B Rossi TM Haworth JC. Celiac disease in a patient with a congenital deficiency of intestinal enteropeptidase. Am J Gastroenterol. (2001) 96:2251–4. 10.1111/j.1572-0241.2001.03970.x 11467662

[12] Wang L Zhang D Fan C Zhou X Liu Z Zheng B Novel compound heterozygous TMPRSS15 gene variants cause enterokinase deficiency. Front Genet. (2020) 11:538778. 10.3389/fgene.2020.538778 33061943 PMC7517701

[13] Polonovski C Laplane R Alison F Navarro J. Pseudo-déficit en trypsinogène par déficit congénital en enterokinase. Etude clinique [trypsinogen pseudo-deficiency caused by congenital enterokinase deficiency. Clinical study]. Arch Fr Pediatr. (1970) 27:677–88. 5488678

[14] Holzinger A Maier EM Bück C Mayerhofer PU Kappler M Haworth JC Mutations in the proenteropeptidase gene are the molecular cause of congenital enteropeptidase deficiency. Am J Hum Genet. (2002) 70:20–5. 10.1086/338456 11719902 PMC384888

[15] Marshall G Mitchell JD Tobias V Messina IM. Arrhythmogenic right ventricular dysplasia in a child with congenital enteropeptidase deficiency and hypogammaglobulinaemia. Aust Paediatr J. (1989) 25:106–8. 10.1111/j.1440-1754.1989.tb01429.x 2735884

[16] Chen CF Yang WC Yang CY Li SY Ou SM Chen YT Urinary protein/creatinine ratio weighted by estimated urinary creatinine improves the accuracy of predicting daily proteinuria. Am J Med Sci. (2015) 349:477–87. 10.1097/MAJ.0000000000000488 25992536

[17] Kitamoto Y Yuan X Wu Q McCourt DW Sadler JE. Enterokinase, the initiator of intestinal digestion, is a mosaic protease composed of a distinctive assortment of domains. Proc Natl Acad Sci U S A. (1994) 91:7588–92. 10.1073/pnas.91.16.7588 8052624 PMC44447

[18] Kitamoto Y Veile RA Donis-Keller H Sadler JE. cDNA sequence and chromosomal localization of human enterokinase, the proteolytic activator of trypsinogen. Biochemistry. (1995) 34:4562–8. 10.1021/bi00014a008 7718557

[19] How KN Leong HJY Pramono ZAD Leong KF Lai ZW Yap WH. Uncovering incontinentia pigmenti: from DNA sequence to pathophysiology. Front Pediatr. (2022) 10:900606. 10.3389/fped.2022.900606 36147820 PMC9485571

[20] Eigemann J Janda A Schuetz C Lee-Kirsch MA Schulz A Hoenig M Non-Skewed X-inactivation results in NF-*κ*B essential modulator (NEMO) *Δ*-exon 5-autoinflammatory syndrome (NEMO-NDAS) in a female with incontinentia Pigmenti. J Clin Immunol. (2024) 45:1. 10.1007/s10875-024-01799-2 39264518 PMC11393190

[21] Juan CK Shen JL Yang CS Liu KL Chen YJ. Flare-up of incontinentia pigmenti in association with behçet disease. J Dtsch Dermatol Ges. (2015) 13:154–6. 10.1111/ddg.12452 25631134

[22] Piccoli GB Attini R Vigotti FN Naretto C Fassio F Randone O NEMO Syndrome (incontinentia pigmenti) and systemic lupus erythematosus: a new disease association. Lupus. (2012) 21:675–81. 10.1177/0961203311433140 22235006

